# Climate change in semi-arid Malawi: Perceptions, adaptation strategies and water governance

**DOI:** 10.4102/jamba.v8i3.255

**Published:** 2016-04-08

**Authors:** Miriam K. Joshua, Cosmo Ngongondo, Fellistus Chipungu, Maurice Monjerezi, Emma Liwenga, Amos E. Majule, Tanya Stathers, Richard Lamboll

**Affiliations:** 1 Department of Geography and Earth Sciences, University of Malawi, Malawi; 2 Ministry of Agriculture, Irrigation and Water Development, Bvumbwe Agriculture Research Station, Malawi; 3 Department of Chemistry, University of Malawi, Malawi; 4 Institute of Resource Assessment, University of Dar es Salaam, Tanzania; 5 Natural Resource Institute, University of Greenwich, United Kingdom

## Abstract

Climate change and variability are a threat to sustainable agricultural production in semi-arid areas of Malawi. Overdependence on subsistence rain-fed agriculture in these areas calls for the identification of sustainable adaptation strategies. A study was therefore conducted in Chikwawa, a semi-arid district in southern Malawi, to: (1) assess community’s perception of a changing climate against empirical evidence, (2) determine their local adaptive measures, (3) evaluate the potential of irrigated agriculture as an adaptive measure in household food security and (4) challenges over access to available water resources. The study employed focus group discussions and key informant interviews to assess people’s perceptions of climate change and variability and their desired interventions. To validate the people’s perceptions, rainfall and temperature data for the period 1960–2010 were analysed. A participatory complete randomised experimental design in both rain-fed and dry season–irrigated conditions was conducted to assess a maize cropping system that would improve adaptation. The study established persistent declining yields from rain-fed production in part because of perennial rainfall failure. In response, the community has shifted its focus to irrigation as an adaptation strategy, which has in turn triggered water conflicts in the community over the control of the resource. Water legislation however fails to adequately provide for rules governing sharing of water resources between various stakeholders. This article therefore recommends development of an appropriate institutional framework that forms a strong basis for equitable distribution of water for irrigation in areas most vulnerable to extreme climate events – including droughts and floods.

## Introduction

### Background

In sub-Saharan Africa, communities significantly depend on subsistence rain-fed agricultural production for food security. However, the heavy reliance on rain-fed agriculture renders many sub-Saharan countries vulnerable to negative consequences of climate change and variability, such as increased occurrence of extreme events such as droughts (Boko *et al*. 2007:433–467; Katz & Brown 1992:300; Parry *et al*. 2004:53–67), contributing to widespread abject poverty and food insecurity. Persistent poverty and food insecurity is closely linked to low and/or declining levels of agricultural productivity (Desanker 2002:3; Irz *et al*. 2001:449–451; Thirtle *et al*. 2001:3). In Malawi, over 90% of the population, mainly comprising resource-poor rural communities, is predominantly engaged in subsistence rain-fed agricultural production. About 60% of this population is classified as food insecure on a year-round basis. Erratic rains across the country have resulted in acute crop failure, despite concerted efforts to improve dissemination of seasonal weather forecasts at the beginning of the rainy season. The failure of crops is associated with food insecurity and also malnutrition, especially among vulnerable rural communities (ActionAid 2006:2; EAD 2002a:125, 2002b:95; Government of Malawi 2006:1–45).

Rural communities are the most vulnerable to the adverse impacts of climate change and variability because of their low adaptive capacity (Adger *et al*. 2003:179–195). Adaptive capacity may be defined as the ability or capacity of a system to modify or change its characteristics or behaviour in order to cope better with existing or anticipated external stresses (Adger *et al*. 2003:179–195). Adaptation to climate change is the adjustment of a natural or human system to moderate the impacts of climate change, to take advantage of new opportunities or to cope with the consequences (Adger *et al*. 2003:179–195). Adaptation is done in response to actual or expected impacts of climate change, which moderates harm or exploits beneficial opportunities (Klein 2001:9).

### Problem statement

Many rural African communities have made adjustments in their traditional ways of life to form adaptation strategies to face the increased interannual climatic variability and occurrence of extreme events (IPCC 2001:398). In Malawi, effective implementation of the various climate change adaptation programmes specifically in subsistence farming is hindered by a number of challenges such as conflicts on access and control of natural resources necessary for adaptation. Identification and supporting appropriate adaptation strategies are therefore vital for ensuring long-term food supplies. The study is premised on the notion that appropriate support is essential and there is ‘no one size fits all’ situation in climate change adaptation. Integrated approach involving local experience and conventional science (including climate and crop science) are also fundamental in generating effective adaptation in farming systems.

#### Objectives

Therefore, the study was aimed at (1) assessing community’s perception of a changing climate against empirical evidence, (2) determining their local adaptive measures, (3) evaluating the significance of irrigated agriculture, as an adaptive measure in the agricultural sector, to household food security and (4) challenges encountered over access and use of available water resources. This study uses a community in Chikwawa District, Southern Malawi, as a case study. This is a semi-arid area that is prone to droughts and floods, with a well-known vulnerability to climate variability and food insecurity (Karanja, Hewitson & Tadross 2004). Specifically, the study reports on climate change risks in the village, impacts of climate change risks on crop production, an analysis of maize yields from rain-fed and irrigated experimental plots and factors affecting successful implementation of irrigation, as an adaptation strategy.

#### Contribution to the field

The study makes a significant contribution to work on adaptation to climate change, both in increasing our understanding of how constrained adaptive capacity affect rural communities and in highlighting the linkages between participatory action research and identification of appropriate adaptation strategies for specific localities. The study is important because many studies agree that climate change remains a threat to rural communities for many decades and call for improving their adaptive capacity. However, limited attention is given to identification and support of adaption strategies suitable for local conditions.

### Literature review

Climate change and variability is a key challenge especially in semi-arid areas of sub-Saharan Africa where peoples’ livelihoods are predominantly dependent on subsistence rain-fed agricultural production (Hulme 1996:104; Hulme *et al*. 2001:145–168; IPCC 1998:1–517, 2001:1–398, 2007:1–104; Martin, Washington & Downing 2000:1473–1479). According to the United Nations Development Programme (UNECA 2007:6), about 70% of Africa’s terrestrial surface is categorised as dry zones. Many parts of Africa, including those deemed to be well endowed with abundant water resources, are already experiencing water stress conditions because of pressures such as rapid population growth (Urama & Ozor 2010:29). Climate change will further compound the already existent water stress conditions. As the water stress conditions intensify, many areas are likely to experience frequent occurrences of water-related conflicts as water and food security is threatened (Brown, Hammill & Mcleman 2007:1141–1154). However, it has been observed that the most vulnerable people to the worst impacts of climate change are those with the least ability to cope with the associated risks (Adger *et al*. 2003:179–195).

In responding to climate change impacts, many least developed countries including Malawi have developed policies and strategies to address climate change–related risks. Examples include National Adaptation Programmes of Actions (NAPAs) that provide a framework of adaptation programmes to ensure rural livelihoods security (Brown *et al*. 2012:1–40; Government of Malawi 2006:1-45; IPCC 2014:151). However, these NAPAs are inadequately designed and implemented (Brown *et al*. 2012:1–40; Joshua, Jalloh & Hachigonta 2014:9). Additionally, National Adaptation Programmes have low local relevance. Climate change impacts can have spatial variations even within one country because of variations in vulnerability context as well as adaptive capacity. This article argues that effective adaptation can occur if interventions fit specific local conditions. Such interventions should therefore ideally be based on location-specific impacts of climate change, involving meaningful participation (that can influence decision making) of local stakeholders and are locally generated. ’Locally generated adaptation approaches and active participation of local actors bring relevance to climate change adaptation programmes’ (Joshua *et al*. 2014:9). It is widely accepted that vulnerable communities have developed various adaptation strategies (Adger *et al*. 2003:179–195; IPCC 2014:151; Tompkins *et al*. 2010:627). Such coping and adaptation strategies are also linked to a very large extent with their perceptions on climate change and its impacts (Kihupi, Chingonikaya & Mahonge 2015:137–147). The strategies include changes in farming calendar, crop varieties and integration of crop and livestock diversification (Action Aid 2006:1–8). However, many rural communities in sub-Saharan Africa have faced challenges in selecting appropriate adaptation strategies in response to climate change impacts including high interannual rainfall variability and unpredictability of rainfall patterns. In the Sahel region, such strategies include adoption of drought-tolerant crops such as sorghum, staggered sowing and use of hand-dug well for irrigation during prolonged drought periods. However, it was noted that the farmers in the Sahel were often not aware of the overall scale of climate change and that their adaptation practices may not be very effective with current climate change impacts, thereby threatening their ability to sustain their family’s livelihood (Deutsche Gesellschaft für Internationale Zusammenarbeit 2011:46). In semi-arid areas of Tanzania, community-based adaptation strategies included fast-maturing crop varieties, buying supplementary foods, increasing wetland cultivation and livestock keeping, water harvesting, practicing mixed cropping and increased emphasis on small stocks (Kangalawe & Lyimo 2013:266–278). The study by Kangalawe and Lyimo (2013:266–278) observed that the vulnerable communities often implemented these adaptation strategies on an ad hoc basis with limited planning. In addition, they further observed that despite their strong potential, if well implemented, there is need for investigating their complementarity as none of these can single-handedly be successful in the adaptation process. Just as in the case of the Sahel, a lack of information, education and a communication strategy on climate change issues affecting rural community livelihoods in the semi-arid areas of Tanzania were also highlighted. This also remains a key policy challenge in many developing countries. The study therefore attempts to address some of these gaps.

## Research methods and design

### Setting

**FIGURE 1:**
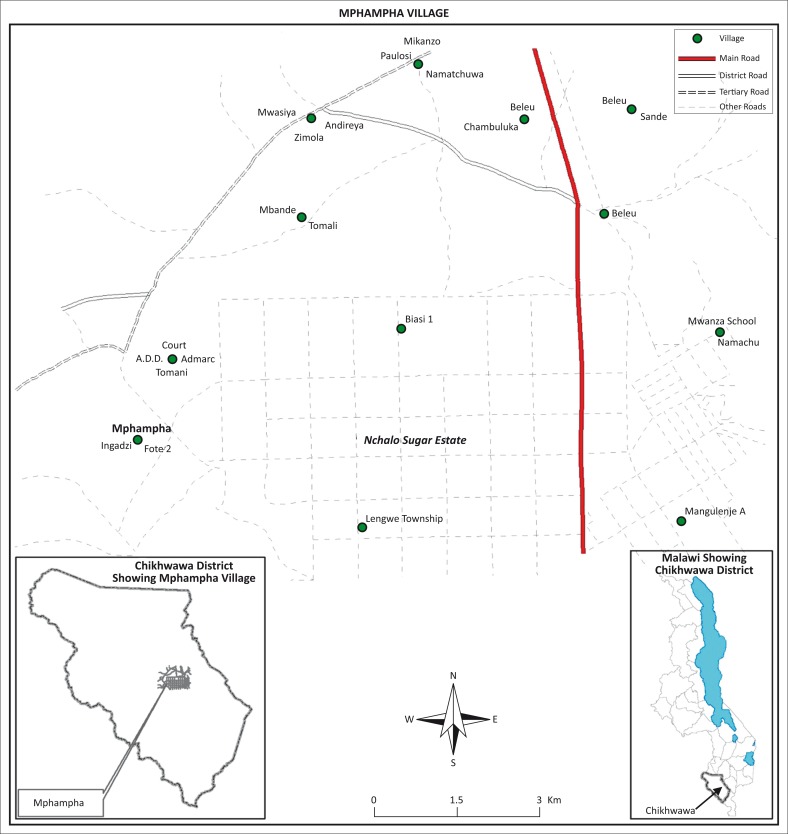
Map of Chikhwawa District in Malawi showing Mphampha Village.

Chikwawa District lies in southern Malawi, a landlocked country south of the equator and bordered by Tanzania to the North and North East, Mozambique to the East, South and South West and Zambia to the West and North West. Mphampha Village is located in Mbewe Extension Planning Area about 27 km from Chikwawa District headquarters (Figure 1). The village is approached along an earth road that branches off the tarmac road to Illovo Nchalo Sugar Estate. On the western side, the village is bordered by the Lengwe National Park.

The study site is generally characterised by a warm tropical savanna climate with distinct wet and dry seasons. The wet season starts in November/December and ends in April/May. The area is located on the leeward side of the Shire Highlands to the East. The annual rains are therefore erratic and highly variable ranging from about 170 mm to 967.6 mm. Temperatures are generally high, with an average annual maximum temperature of 37.6 °C, occurring in December and a minimum 27.6 °C, occurring in July every year. Monthly mean temperatures are usually above 20 °C.

### Data collection and analysis

The study used both qualitative and quantitative methods. Participatory Rural Appraisals, employing focus group discussions and key informant interviews, were used to assess people’s perceptions of climate change and variability and their desired interventions. Rainfall and temperature data for stations within the Extension Planning Area, as well as historical records of natural droughts and flood events, were also analysed. This allowed a comparison of the people’s perceptions with the actual climate pattern for the area. A complete randomised experimental design was conducted, with the participation of the villagers, to assess a maize cropping system that would improve adaptation. The trials were conducted in both rain-fed and dry season–irrigated conditions. Demonstration plots employing complete randomised block design of maize crop, a staple for the district were set for three consecutive growing seasons (2008/2009, 2009/2010 and 2010/2011).

Evaluations were done for the selection of maize variety and seedbed preparation method. The study used maize varieties SC 403 and DK 8033 for the first 2 years, and in the third year ZM 309 was added to the pool of varieties to be studied. The maize was planted using traditional flat and tie ridges for rain-fed production and sunken beds for irrigated maize. Yield was then compared for the two farming systems.

Daily maximum and minimum temperature and rainfall data at Chikwawa, Ngabu, Nchalo and Makhanga Stations for the period 1960–2010 were obtained from the Malawi Department of Climate Change and Meteorological Services. These were analysed for evidence of climate change using the nonparametric Mann–Kendall Statistic (Kendall 1975:160; Mann 1954:245–259) as recommended by the World Meteorological Organisation. The Mann–Kendall Test attaches significance to either a positive or negative trend in a variable and a 95% confidence interval was adopted in this study.

## Results and discussions

### Community perceptions on climate, climate change and indicators

The climate of Mphampha Village is characterised by hot, dry conditions with low annual rainfall and number of rainy days. The normal rainy season lasts from November to March. However, part of the rainy season is characterised by very strong winds which destroy crops and houses.

**FIGURE 2:**
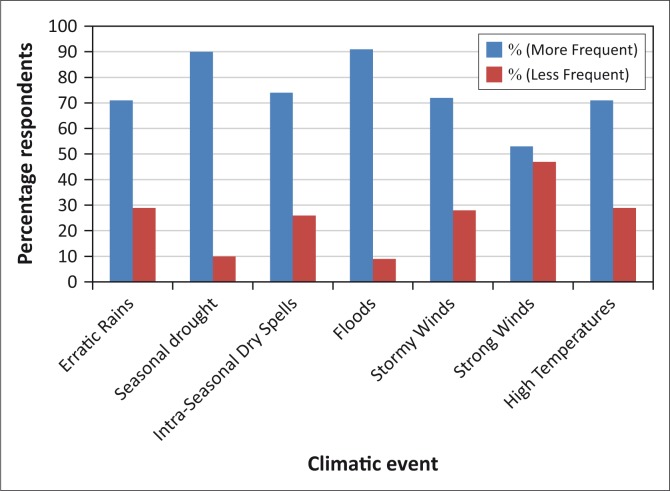
Aspects of climate that have changed and frequency – from household respondents.

The community perceived that the area was now experiencing variations in climate (Figure 2). The key indicators reported were high rainfall variability, characterised by unpredictable seasonal rainfall pattern, warmer days and cooler nights (Figure 2). In terms of rainfall, the villagers reported that the area had also been experiencing variable, but late onset of rains, with the onset shifting from November to December. However, the rainfall season has become shorter as the late onset is more often followed by early cessation of rainfall. The amount of rainfall is also perceived to have declined because of frequent prolonged dry spells and droughts experienced in the course of the wet season (Figure 2). The people also observed that the midseason drought has become an annual event in the past 5 years. The area can therefore experience a good onset of rains followed by a prolonged dry spell, for example, for a month (Figure 2). The rains pick up towards the end when food crops such as maize had already wilted, hence leading to low yield. Occurrence of strong winds and cold nights during the rainy season also affect the rainfall pattern adversely. Strong winds are also known to drive away clouds, whereas low temperatures reduce evaporation as well as rainfall amounts.

The people also observed that occurrence of floods in the area has recently become rare (Figure 2). The few episodes reported were mainly driven by upland runoff derived from heavy rainfall thereof. However, some of the villagers benefited from floods for agricultural production. After such floods, the villagers could register higher yields from the top soils, washed from uplands and residual moisture after the floodwaters recede. Reduced floods have therefore increased their vulnerability in terms of household food security.

Tables 1 and 2 provide summaries of major climatic events that had occurred in Mphampha Village since 1970s and associated impacts based on people’s experiences.

**TABLE 1:** Major climatic events and their associated impacts in the village.

Year	Events		Impacts	
	
	Women	Men	Women	Men
1986/1987	-	Floods	-	Famine - Fields and crops washed away
1991/1992	Drought	Drought	Famine	No yield, people relied on rice
1993/1994	Drought	-	Famine	-
1997/1998	Floods	Floods (because of high rains)	People killed, houses and crops destroyed	Crops destroyed and people killed
2002	Drought	Strong winds	Famine	Destroyed houses
2005/2006†	Floods	Drought	Low yield	Low yield
2007/2008–2012	Prolonged dry spells and midseason drought	Prolonged dry spells and midseason drought; at tasseling stage (maize)	Low or no harvest	Low yield

† The area received little rains but was heavily affected by floods – resulting from heavy rains from upland areas.

**TABLE 2:** Impacts of climate change and variability in Mphampha Village – from key informant’s perspective.

Year	Event	Impact
1970s	Too little rainfall	Crops died; people who had money bought rice from Blantyre
1984	Too little rainfall	People survived on finger millet
1994	Too little rainfall	Crop failure, people survived on finger millet
2001	Erratic rainfall – rains just came twice throughout the season	Starvation, people survived on maize husks (*madeya*); many goats died
2006	Too much rainfall	Crops were damaged and harvest was poor

The perceptions of the community were compared with historical records of major climatic events (Tables 1 and 2). The perceptions of the people were in general agreement with the historical record. For instance, the observations on increase in occurrence of climatic extremes leading to disasters agree with figures for Malawi as shown in Table 3.

**TABLE 3:** Top 10 natural disasters in Malawi for the period 1900–2012 sorted by numbers of total affected people.

Disaster	Date	Total number of people affected
Drought	April 1992	7 000 000
Drought	October 2005	5 100 000
Drought	February 2002	2 829 435
Drought	February 1990	2 800 000
Drought	1987	1 429 267
Drought	October 2007	520 000
Flood	January 2001	500 000
Flood	18 February 1997	400 000
Flood	28 December 2002	246 340
Flood	18 November 2007	180 246

*Source*: Guha-Sapir, D., Below, R. & Hoyois, Ph., *EM-DAT: The CRED/OFDA International Disaster Database*, Université Catholique de Louvain, Brussels, Belgium, viewed 22 March 2012, from http://www.emdat.be/country_profile/index.html

The records also showed that the onset of planting rains was variable, but increasingly late, which is in agreement with the people’s perceptions. For instance, the planting rains were recorded on December 15 for the 2009/2010 season, first week of January for the 2010/2011 season and 11 November for the 2011/2012 season. For all three seasons the rains were nevertheless highly erratic and poorly distributed.

Comparison of the observations with empirical evidence using temperature and rainfall data for nearby climate stations at Chikwawa, Ngabu, Makhanga and Nchalo also showed some similarities. Daily rainfall (1978–2008) and temperature data (1971–2008) for three stations in the area, namely Nchalo, Makhanga and Ngabu, were analysed using standard techniques. The Mann–Kendall statistics for the stations are shown in Table 4.

**TABLE 4:** Mann–Kendall trends in climatic variable.

Variable or station	*a*	Nchalo	Chikwawa	Makhanga	Ngabu	*b*
Daily minimum temperature	-1.96	13.52	n/a	4.56	1.49	1.96
Daily maximum temperature	-1.96	9.62	n/a	4.85	-1.22	1.96
Daily temperature Range	-1.96	-3.01	n/a	-0.62	-4.64	1.96
Monthly minimum temperature	-1.96	3.7	n/a	1.68	0.51	1.96
Monthly maximum temperature	-1.96	2.24	n/a	0.91	-0.24	1.96
Monthly temperature range	-1.96	-1.03	n/a	-0.15	-0.88	1.96
Daily rain	-1.96	-1.4	-2.29	0.52	-1.66	1.96
Monthly rain	-1.96	-1.15	-0.62	-0.06	-1.34	1.96

*a* and *b* indicate limits of the 95% confidence interval.

From Table 4, daily minimum temperatures have increased in the area of which Nchalo and Makhanga Stations had significant increases at 95%. Daily maximum temperatures also increased significantly at Nchalo and Makhanga Stations, whereas a local decrease in the daily maximum is suggested for Ngabu Station. The diurnal temperature experienced a decrease, which is a key indication that suggests that the minimum temperatures increased more than the maximum temperatures. Significant increases in the diurnal temperature range were observed at Ngabu and Nchalo Stations. At monthly level, minimum mean temperatures increased with Nchalo reporting the only significant trends. Monthly maximum mean temperatures increased significantly at Nchalo and Makhanga with the former reporting significant increases, whereas a statistically insignificant decrease is suggested for Ngabu. Just like the daily time scale, the temperature range also decreased at monthly scales at all sites, albeit insignificantly. The area mostly experienced a decrease in daily rainfall, with Chikwawa Boma Station experiencing a significant decrease at 95% levels. Although the monthly rainfalls also suggest decreased trends, none of the stations experienced a significant trend at 95% level. We, therefore, suggest that people’s perception of changes in rainfall and temperature generally agree with the empirical evidence. Similar comparisons are evident in Table 3. It is worth noting that 9 of the top 10 natural disasters from 1900 to 2012 have occurred between 1990 and 2007, indicating high frequency in less than 20 years. In contrast, a similar study in Zimbabwe showed ‘an overall mismatch in the farmers’ perceptions and the evidence from the meteorological data’ specifically with regards to change in rainfall trends (Moyo *et al*. 2012:333). Despite this, farmers’ perceptions on temperature trends and seasonal distribution of rainfall agreed with empirical data (Moyo *et al*. 2012: 317–333) and support this study’s conclusions.

#### Impact of climate change and variability on crop production

The major food crops grown in the area in order of their priority are sorghum, maize, cowpeas, millet, and vegetables. Other food crops grown are sweet potatoes and cassava, whereas cotton is the major cash crop followed by vegetables and cowpeas.

Climate change is impacting on the yields of these crops as well as farming practices in the village, with yields declining per unit area. Maize and other food crops such as rice, pigeon peas, and vegetables such as pumpkin leaves are being affected because of prolonged drought and inadequate rainfall. Consequently, a number of practices have been abandoned following no or poor crop yield. The practices include:

- brewing of free beer for the whole village
- children’s games (*nomi*/*masanje*), where they cook leftovers collected from the fields after main harvest
- *suluma*/*ndidze* or *chipako*, games for physical fitness (because of famine as bodies are weak)
- local maize production in favour of hybrid varieties.

**FIGURE 3:**
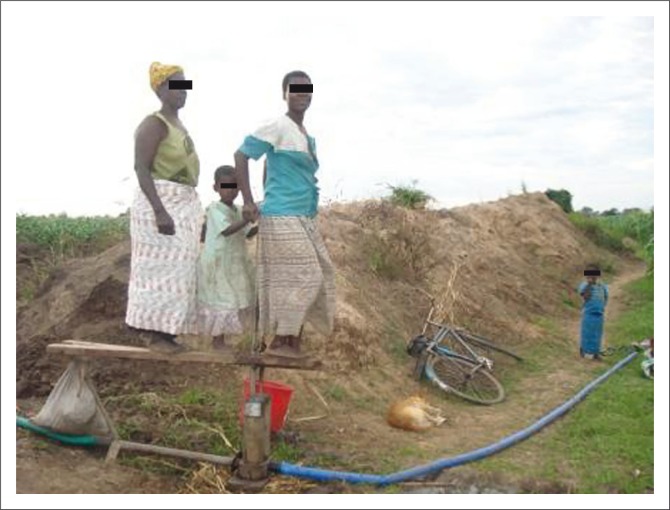
Women irrigating maize in Mphampha Village.

The cropping calendar for all the crops has experienced changes because of changes in rainfall pattern (Table 5). Farm activities under the calendar are largely dictated by the onset of rainfall and distribution. Because of the changes experienced, staggered planting is also becoming a common practice in the village. Farmers can in some years plant up to five times until rains are stable. Additionally, following the recent climatic events, for the last 10 years, rain-fed maize production is on the decline, whereas the acreage of cotton and sorghum is on the rise. Farmers reported persistent declining rain-fed maize yield and high yield for irrigated maize. Maize production is mainly done under treadle pump irrigation (Figure 3), with irrigated area per household ranging from 0.1 to 0.2 acres. These changes have been necessitated as a response to the current changes in the yield/acre (productivity) of the recent cropping years.

**TABLE 5:** Seasonal calendar for Mphampha Village.

Activity	Rain-fed Maize and Sorghum		Cotton	
	
	Original period	New period	Original period	New period
Farm preparation	July–October	July–October	July–October	July–October
Planting	November–December	November–December depending on rainfall onset; sometimes it can be done January	November–December	November–December (sometimes December–January)
Weeding	November–December	January–March	November–February	January–March
Spraying	n/a	n/a	December–April	January–February
Harvesting	April–May	April	April–June	April–June
Processing and grading	June–August	-	April–June	May–June

#### Learning plots evaluation

**FIGURE 4:**
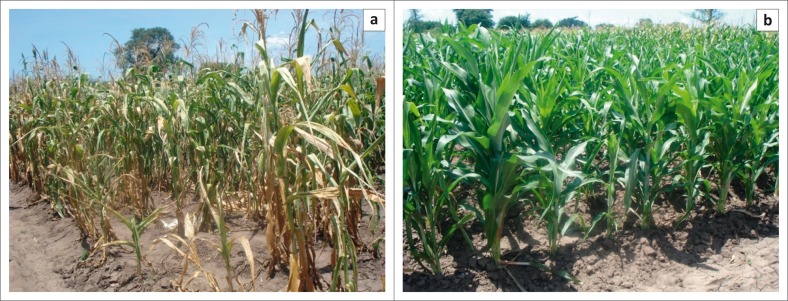
Rain-fed plot (a) and another rain-fed learning plot (b) planted a month later during the 2008/2009 season in Mphampha Village.

The results from the learning plots and farmers’ gardens showed a complete crop failure for the 2008/2009 and 2009/2010 growing seasons. In the first experimental year, rain-fed maize performed fairly well on tied ridges despite the prolonged dry spells experienced. Although, there was no yield because of late planting, the learning plot crop stand was healthier than nearby gardens (Figure 4). In the third year (2010/2011), farmers did not register any yield, whereas some yield was nevertheless obtained from the experimental plots. In 2011, the highest maize yield under rain-fed production was 0.8 t per ha from box ridges with manure and inorganic fertilisers. The low-yielding but early-maturing variety, SC 403 yielded 0.7 t per ha, which was higher than the late-maturing but high-yielding variety, DK 8033 which provided 0.05 t per ha. Overall average yield performance showed that tie ridges was the best tillage method for seedbed preparation, whereas flat cultivation recorded the lowest yield of maize. Variety evaluation showed higher yields for the maize varieties ZM 309 and SC 403 than DKC 8033. The maize yield almost doubled with manure application combined with inorganic fertiliser, which is attributable to water conservation properties of manure which helped the crop during prolonged dry spells, in addition to provision of nutrients. This result concurs with Achieng *et al*. (2010:436), who reported that farmyard manure from a combination of cow dung and urine provide a balanced nutrition to plants and hence enhance crop productivity. Reduction in maize yield is mainly attributed to midseason drought or prolonged dry spells (Table 5) and increasing prevalence of the crop pests. The communities also associate proliferation of pests with impacts of climate change or variability such as high temperatures and very low rainfall. The prevalent crop pests in the village include stalk borers (*mphutsi*), millipedes (*bongololo*) and lice (*nsabwe*). Stalk borers and millipedes affect maize especially at the development stage. However, chemicals (such as cypermethrine) are not locally available.

The study noted that the availability of water, especially at tasseling stage, is the main factor that limited maize yields.

For instance, in the 2009/2010 growing season, the village started registering high yield from irrigation plots. This resulted from International Development Research Centre (IDRC)–funded climate change adaptation project’s intervention which involved empowering traditional leaders to negotiate with a local sugar company through the District Executive Committee for increased water supply in the outflow channel. Therefore, under irrigation, the maize variety DK 8033 provided 6.15 t per ha in 115 days, but required more water than the variety SC 403 which provided 5.0 t per ha in 95 days. Average household size is six persons with four children per household. Maize requirement for household food security is 600 kg. Average land holding size is 0.2 ha. This shows that households will manage to obtain a yield of 935 kg from their 0.2 ha of land. Thus promoting irrigated maize production in the village may ensure food security for the whole year and surplus for sale. In 2011, the highest maize yield under rain-fed production was 0.8 t per ha from box ridges with manure and inorganic fertilisers. The low-yielding, but early-maturing variety, SC 403 yielded 0.7 t per ha, which was higher than the late-maturing but high-yielding variety DK 8033, which provided 0.05 t per ha.

### Water governance challenges

Main water source for irrigation is an outflow channel from a local sugar plantation. However, there are water governance problems between the sugar company and the villagers. This relates to differences in bundle of rights held by each actor. The villagers have limited access and use rights to the water in the outflow channel and no management rights. In contrast, the sugar company holds all the three sets of rights supported by the *Water Resources Act* (Government of Malawi 2013:29–32). Hence, the company can control the timing and amount of water drained from the plantation into the outflow channel without consultation with villagers. Because of this, the company would stop releasing water into the outflow channel at a time when the maize crop was at tasseling stage – leading to crop failure and consequently further household food insecurity.

For 3 years from 2008 to 2011, the villagers registered high maize yield with the assistance of Evangelical Association of Malawi and the IDRC-funded Climate Change adaptation–supported project. The project intervened in the supply of water which was initially being cut off when maize was at the tasseling stage. For 3 consecutive years the company provided water by letting it flow throughout the growing season and deepening the channel. However, support reduced or stopped when the project came to an end in 2010/2011. This is probably because water sources are inadequate because of increased water demand – for irrigation. Further, the Water Resources legislation (Government of Malawi 2007:1–21, 2013:1–83) provides no clear guidance with regards to such water transfer arrangements. For example, section 65 of the *National Water Resources Act* (Government of Malawi 2013:39) (inserted below) does not include licence holders who abstract water from water courses for commercial purposes. Hence, they lack mandate to include the arrangement in their water management plans and local government has no power to direct water users to such water management arrangements:

65.__…

(2) If the Minister, on the advice of the Authority, is satisfied that, by reason of an exceptional shortage of rain or by reason of accident or other unforeseen circumstances, a serious deficiency of water for essential domestic purposes exists or is threatened in any area, he may, by order__

(*a*) declare that an emergency exists; and

(*b*) direct a person who has a supply of water in excess of his needs for domestic purposes to supply to the area concerned, or to a specified person in the area, such quantity of water, and for such period, as the order may specify. (p. 39)

There is a general consensus that irrigation should be a means of increasing adaptation to Climate Change in both villages. However, this should be approached carefully as it calls for innovative ways – both technical and institutional – on how often scarce water resources are managed. Further accessibility to water resources is not uniform as sources of water in the village vary. The outflow channel constructed by the sugar company for the village provided tangible solutions for agricultural production under the semi-arid conditions. To sustain food production therefore calls for governments’ review of the water legislation. This includes clarification of operational rules for commercial water users in response to subsistent farmers’ irrigation challenges associated with climate risks. Additionally, there is a need for intervention in negotiations with the company. A possible solution is for Malawi government to partner with the company to ensure continuous supply of water to the village and on a wider scale, to cover more farmers. This will be in line with international agreements on effective water governance. For example, the UN 2000 Millennium Assembly emphasised conservation and stewardship in protecting our common environment and especially *to stop the unsustainable exploitation of water resources, by developing water management strategies at the regional, national and local levels, which promote both equitable access and adequate supplies*. This was endorsed at the World Summit on Sustainable Development in 2002 where Heads of State agreed a specific target *to prepare IWRM and water efficiency plans by 2005*. Given the complexities of water use within society, developing, allocating and managing it *equitably* and *efficiently* and *ensuring environmental* sustainability requires that the disparate voices are heard and respected in decisions over common waters especially with regards to operational rules. Operational rules specify what resource users are constrained, allowed and expected to do (Ostrom 1990:52, 2002:1332).

Furthermore, the study area experiences flooding almost every rainfall season; hence, there is high potential to meet irrigation water requirements for the community through rain water harvesting. However, the community has limited capacity for water harvesting. This study therefore recommends that relevant stakeholders including government should consider enhancing this intervention through use of simple technologies such as earth dams.

In addition, there is need to reduce water use demand for irrigation through supporting of other local desired interventions that are not directly linked to irrigation because climate projections and water demand for the study area suggest increased water shortages or water stress by 2025 (Southern African Development Community 2005:6). These interventions include external support on improved sorghum and livestock production and links to profitable markets. Alternatively, there is need to build farmers’ capacity on climate SMART agriculture, an integrative approach to address interlinked challenges of food security and climate change. The approach may therefore be applied in the area through promoting conservation of water, for example, tower gardening, or provide shared tariffs between the sugar company and farmers to enhance the sense of responsibility and reduce water use by farmers. This could apply agreed monetary or nonmonetary tools. In essence, this implies that ‘the consumer will change his or her consumption preferences. Regulatory instruments that involve permits, restrictions, and allocations to various users and uses can also reduce water demand’ (Global Water Partnership 2003:23) or ensure sustainable and equitable use of water. However, these instruments should not exclude the poor farmers from benefiting from the resource. Development of the instruments can be done in a participatory approach involving all the relevant stakeholders including the local farmers, local sugar company and state actors.

## Conclusion

The study shows that adaptation to climate change and climate variability remains a challenge in many semi-arid areas using villages in Chikwawa District in Malawi as a case study. The district is a semi-arid, drought and flood-prone area in southern Malawi. The study has revealed that the area is experiencing interannual climate variability and the effects of climate change and climate variability. People’s perceptions suggest that winds are becoming stronger, daily temperatures are warming, rainfall amount is declining and its pattern has become unpredictable and unreliable. In addition, dry spells within the rain season are also becoming very frequent and prolonged. These observations agree with empirical scientific data. These changes in the climate regimen have affected members of the community’s livelihood strategies, especially in agriculture.

Irrigation is seen as a major adaptation strategy in addition to others, including livestock production. However, the villagers have limited capacity to improve their adaptive capacity. For example, farmers have intensified production of drought-resistant crops such as sorghum, but have limited access to good seed and profitable market. Access to irrigation water is more preferred, although it is presently limited because of water governance or latent conflicts. Additionally, the strategy is threatened by future water demand projections. Livestock production, particularly of resilient types such as goats and local chickens, offer a better adaptive strategy. However, farmers have limited access to improved livestock breeds. Most of the challenges being faced by the farmers as a result of climate change and variability can be addressed if the relevant policy is developed and/or enforced and relevant irrigation infrastructure is provided. The local governments, however, have inadequate legal support to operate in their local areas.

## References

[CIT0001] Achieng J.O., Ouma G., Odhiambo G. & Muyekho F, 2010, ‘Effect of farmyard manure and inorganic fertilizers on maize production on Alfisols and Ultisols in Kakamega, western Kenya’, *Agriculture and Biology Journal of North America* 1(4), 430–439.

[CIT0002] ActionAid, 2006, *Climate change and smallholder farmers in Malawi: Understanding poor people’s experiences in climate change adaptation*, viewed 10 December 2014, from https://www.actionaid.org.uk/sites/default/files/doc_lib/malawi_climate_change_report.pdf

[CIT0003] Adger W.N., Saleemul Huq S., Brown K., Conway D. & Hulme M, 2003, ‘Adaptation to climate change in the developing world’, *Progress in Development Studies* 3, 179–195.

[CIT0004] Boko M., Niang I., Nyong A., Vogel C., Githeko A., Medany M. et al., 2007, ‘Africa. Climate change 2007: Impacts, adaptation and vulnerability’, in Parry M.L., Canziani O.F., Palutikof J.P., van der Linden P.J. & Hanson C.E. (eds.), *Contribution of Working Group II to the Fourth Assessment Report of the Intergovernmental Panel on Climate Change*, pp. 433–467, Cambridge University Press, Cambridge, UK.

[CIT0005] Brown O., Hammill A. & Mcleman R, 2007, ‘Climate change as the “new” security threat: Implications for Africa’, *International Affairs* 83(6), 1141–1154.

[CIT0006] Brown D., Rance Chanakira R., Chatiza K., Dhliwayo M., Dodman D., Masiiwa M. et al., 2012, *Climate change impacts, vulnerability and adaptation in Zimbabwe*, IIED Climate Change Working Paper, International Institute for Environment and Development, London, UK, pp. 1–40.

[CIT0007] Desanker P.V, 2002, *Impact of climate change on Africa*, WWF Climate Change Program, pp. 1–6, viewed 10 December 2014, from http://www.panda.org/climate

[CIT0008] Deutsche Gesellschaft für Internationale Zusammenarbeit (GIZ), 2011, *Adaptation to climate change with a focus on rural areas and India*, Ministry of Environment and Forests, Government of India, New Dehli, pp. 44–82.

[CIT0009] Environmental Affairs Department (EAD), 2002a, *Initial National Communication under the United Nations Framework Convention on Climate Change*, Ministry of Natural Resources and Environmental Affairs, Lilongwe, Malawi, pp. 1–125.

[CIT0010] Environmental Affairs Department (EAD), 2002b, *The vulnerability and adaptation assessment report of 2001*, Department of Environmental Affairs, Lilongwe, Malawi, p. 95.

[CIT0011] Global Water Partnership, 2003, *Effective water governance*, Global Water Partnership Secretariat, Stockholm.

[CIT0012] Government of Malawi, 2006, *National Adaptation Programme of Action (NAPA)*, Ministry of Natural Resources and Environmental Affairs, Lilongwe, Malawi, pp. 1–45.

[CIT0013] Government of Malawi, 2007, *National water policy*, 2nd edn., Ministry of Irrigation and Water Development, Lilongwe, Malawi, pp. 1–21.

[CIT0014] Government of Malawi, 2013, *Water Resources Act*, Ministry of Irrigation and Water Development, Lilongwe, Malawi, pp. 1–83.

[CIT0015] Guha-Sapir D., Below R. & Hoyois Ph, *EM-DAT: The CRED/OFDA International Disaster Database*, Université Catholique de Louvain, Brussels, Belgium, viewed 22 March 2012, from http://www.emdat.be/country_profile/index.html

[CIT0016] Hulme M. (ed.), 1996, *Climate change in Southern Africa: An exploration of some potential impacts and implications in the SADC region*, Climatic Research Unit, University of East Anglia, Norwich, UK, pp. 1–104.

[CIT0017] Hulme M., Doherty R., Ngara T., New M. & Lister D, 2001, ‘African climate change: 1900–2100’, *Climate Research* 17, 145–168.

[CIT0018] IPCC, 1998, ‘The regional impacts of climate change: An assessment of vulnerability’, in Watson R.T., Zinyowera M.C. & Moss R.H. (eds.), *Special report of IPCC Working Group II*, pp. 1–517, Cambridge University Press, Cambridge, UK.

[CIT0019] IPCC, 2001, ‘Climate change 2001: Synthesis report’, in Watson R.T. and the Core Writing Team (eds.), *Contribution of Working Groups I, II, and III to the Third Assessment Report of the Intergovernmental Panel on Climate Change*, pp. 1–398, Cambridge University Press, Cambridge, UK.

[CIT0020] IPCC, 2007, ‘Climate change 2007: Synthesis report’, in Core Writing Team, Pachauri R.K. & Reisinger A. (eds.), *Contribution of Working Groups I, II and III to the Fourth Assessment Report of the Intergovernmental Panel on Climate Change*, pp. 1–104, IPCC, Geneva, Switzerland.

[CIT0021] IPCC, 2014, ‘Climate change 2014: Synthesis report’, in Core Writing Team, Pachauri R.K. & Meyer L.A. (eds.), *Contribution of Working Groups I, II and III to the Fifth Assessment Report of the Intergovernmental Panel on Climate Change*, pp. 1–151, IPCC, Geneva, Switzerland.

[CIT0022] Irz X., Lin L., Thirtle C. & Wiggins S, 2001, ‘Agricultural productivity growth and poverty alleviation’, *Development Policy Review* 19(4), 449–466.

[CIT0023] Joshua M., Jalloh A. & Hachigonta S, 2014, *Review of research and policies for climate change adaptation in urban areas in southern Africa*, viewed 30 November 2014, from http://www.future-agricultures.org/publications/research-and-analysis/working-papers/1907-review-of-research-and-policies-for-climate-change-adaptation-in-urban-areas-in-southern-africa

[CIT0024] Kangalawe R.Y.M. & Lyimo J.G, 2013, ‘Climate change, adaptive strategies and rural livelihoods in semiarid Tanzania’, *Natural Resources* 4, 266–278.

[CIT0025] Karanja F.K., Hewitson B. & Tadross M, 2004, *Climate change scenarios and vulnerability assessments for selected countries in Eastern and Southern Africa*, UNEP Contribution Paper No.2, viewed 30 November 2014, from http://www.unep.org/themes/climatechange/PDF/Paper_No.2.pdf

[CIT0026] Katz R.W. & Brown B.G, 1992, ‘Extreme events in a changing climate: Variability is more important than averages’, *Climatic Change* 21, 298–302.

[CIT0027] Kendall M.G, 1975, *Rank correlation methods*, 4th edn., Charles Griffin, London, p. 160.

[CIT0028] Kihupi M.L., Chingonikaya E.E. & Mahonge C, 2015, ‘Smallholder farmers’ perception of climate change versus meteorological data in semi-arid areas of Iringa district, Tanzania’, *Journal of Environment and Earth Science* 5(2), 137–147.

[CIT0029] Klein R.J.T, 2001, *Adaptation to climate change in German official development assistance: An inventory of activities and opportunities, with a special focus on Africa*, Deutsche Gesellschaft für Technische Zusammenarbeit, Eschborn, Germany, p. 42.

[CIT0030] Mann H.B, 1954, ‘Non-parametric test against trend’, *Econometrica* 13, 245–259.

[CIT0031] Martin R.V., Washington R. & Downing T, 2000, ‘Seasonal maize forecasting for South Africa and Zimbabwe derived from an agroclimatological model’, *Journal of Applied Meteorology* 39, 1473–1479.

[CIT0032] Moyo M., Mvumi B.M., Kunzekweguta M., Mazvimavi K., Craufurd P. & Dorward P, 2012, ‘Farmer perceptions on climate change and variability in semi-arid Zimbabwe in relation to climatology evidence’, *African Crop Science Journal* 20, 317–335.

[CIT0033] Ostrom E, 1990, *Governing the commons*, Cambridge University Press, New York, pp. 1–271.

[CIT0034] Ostrom E, 2002, ‘Common-pool resources and institutions: Toward a revised theory’, in Garnder B. & Rausser G. (eds.), *Handbook of agricultural economics*, pp. 1317–1339, Elsevier Science B.V., North Holland

[CIT0035] Parry M.L., Rosenzweig C.A., Iglesias A., Livermore M. & Fisher G, 2004, ‘Effects of climate change on global food production under SRES emissions and socioeconomic scenarios’, *Global Environmental Change* 14, 53–67.

[CIT0036] Southern African Development Community, 2005, *Southern African Development Community Regional water policy*, viewed 12 October 2014, from http://www.sadc.int/documents-publications/show/Regional_Water_Policy.pdf

[CIT0037] Thirtle C., Irz X., Wiggins S., Lin L. & McKenzie-Hill C, 2001, *Relationship between changes in agricultural productivity and the incidence of poverty in developing countries*, DFID Report No. 7946 27/02/2001, pp. 1–33.

[CIT0038] Tompkins E.L., Adger W.N., Boyd E., Nicholson-Cole S., Weatherhead K. & Arnell N, 2010, ‘Observed adaptation to climate change: UK evidence of transition to a well-adapting society’, *Global Environmental Change* 20, 627–635.

[CIT0039] United Nations Economic Commission for Africa (UNECA), 2007, ‘Africa review report on drought and desertification’, *Fifth Meeting of the Africa Committee on Sustainable Development (ACSD-5) Regional Implementation Meeting (RIM) for CSD-16*, Addis Ababa, October 22–25, 2007, pp. 1–65.

[CIT0040] Urama K.C. & Ozor N, 2010, *Impacts of climate change on water resources in Africa: The role of adaptation*, pp. 1–29, viewed 30 November 2014, from http://www.ourplanet.com/climate-adaptation/Urama_Ozorv.pdf

